# Wall of Resilience: How the Intestinal Epithelium Prevents Inflammatory Onslaught in the Gut

**DOI:** 10.1016/j.jcmgh.2024.101423

**Published:** 2024-10-24

**Authors:** Eva Liebing, Susanne M. Krug, Markus F. Neurath, Britta Siegmund, Christoph Becker

**Affiliations:** 1 Department of Medicine 1, Universitätsklinikum Erlangen, Friedrich-Alexander-Universität Erlangen-Nürnberg, Erlangen, Germany; 2 Deutsches Zentrum Immuntherapie, Erlangen, Germany; 3 Clinical Physiology/Nutritional Medicine, Charité–Universitätsmedizin Berlin corporate member of Freie Universität Berlin and Humboldt-Universität zu Berlin, Berlin, Germany; 4 Department of Gastroenterology, Infectious Diseases and Rheumatology, Campus Benjamin Franklin, Charité–Universitätsmedizin Berlin corporate member of Freie Universität Berlin and Humboldt-Universität zu Berlin, Berlin, Germany

**Keywords:** Intestinal Epithelium, Barrier Dysfunction, Inflammatory Bowel Diseases

## Abstract

The intestinal epithelium forms the boundary between the intestinal immune system in the lamina propria and the outside world, the intestinal lumen, which contains a diverse array of microbial and environmental antigens. Composed of specialized cells, this epithelial monolayer has an exceptional turnover rate. Differentiated epithelial cells are released into the intestinal lumen within a few days, at the villus tip, a process that requires strict regulation. Dysfunction of the epithelial barrier increases the intestinal permeability and paves the way for luminal antigens to pass into the intestinal serosa. Stem cells at the bottom of Lieberkühn crypts provide a constant supply of mature epithelial cells. Differentiated intestinal epithelial cells exhibit a diverse array of mechanisms that enable communication with surrounding cells, fortification against microorganisms, and orchestration of nutrient absorption and hormonal balance. Furthermore, tight junctions regulate paracellular permeability properties, and their disruption can lead to an impairment of the intestinal barrier, allowing inflammation to develop or further progress. Intestinal epithelial cells provide a communication platform through which they maintain homeostasis with a spectrum of entities including immune cells, neuronal cells, and connective tissue cells. This homeostasis can be disrupted in disease, such as inflammatory bowel disease. Patients suffering from inflammatory bowel disease show an impaired gut barrier, dysregulated cellular communication, and aberrant proliferation and demise of cells. This review summarizes the individual cellular and molecular mechanisms pivotal for upholding the integrity of the intestinal epithelial barrier and shows how these can be disrupted in diseases, such as inflammatory bowel disease.

**Summary**

This review delineates the structure of the intestinal epithelium with its cellular and molecular organization, cell death and cell regeneration, and communication with surrounding cells and the microbiome, spotlighting disruptions in inflammatory bowel disease within concise detail.

Over the past decade, research has shown that the complex interplay of the intestinal mucosa with the immune system and the microbiome is necessary to maintain proper intestinal homeostasis. The intestinal epithelium has cellular and molecular means of communication through which it interacts with the microbiota and the immune system. Unfortunately, this delicate equilibrium is susceptible to disruption by genetic predisposition, microbial composition, or other environmental factors. Such effects favor barrier dysfunctions that, as a secondary effect, can promote the development of diseases, such as inflammatory bowel disease (IBD). IBD is a chronic, recurrent inflammation of the intestinal tract, the cause of which is believed to be multifactorial. It is currently believed that the disease occurs in individuals with a genetic predisposition and a dysregulated immune response to the intestinal microbiota.^1^ The 2 types of IBD are ulcerative colitis (UC) and Crohn's disease (CD). These diseases are seen in all age groups and are considered to be Western diseases, although the incidence rates in developing countries are increasing.^2^, ^3^, ^4^ Furthermore, there is a subgroup known as very early onset IBD, which affects children younger than 6 years of age. These young patients face a severe condition and often do not respond to conventional therapies, making customized treatments essential for their care.^5^ Through its various cell types and their communication with the microbiota and immune cells, its cell-cell contacts, and the regulation of paracellular exchange, the intestinal epithelium is a true master of maintaining tissue integrity and defense. This review provides a comprehensive overview of the cellular and molecular composition of the intestinal epithelium, and its communication with other cells of the gastrointestinal tract in efforts to maintain intestinal homeostasis, but also provides insight into the mechanisms for dysfunction of this barrier in IBD pathogenesis.

## The Cellular Components of the Intestinal Epithelium and their Roles in Maintaining Barrier Homeostasis

### Homeostatic Intestinal Stem Cells and their Niche

**Figure 1:**
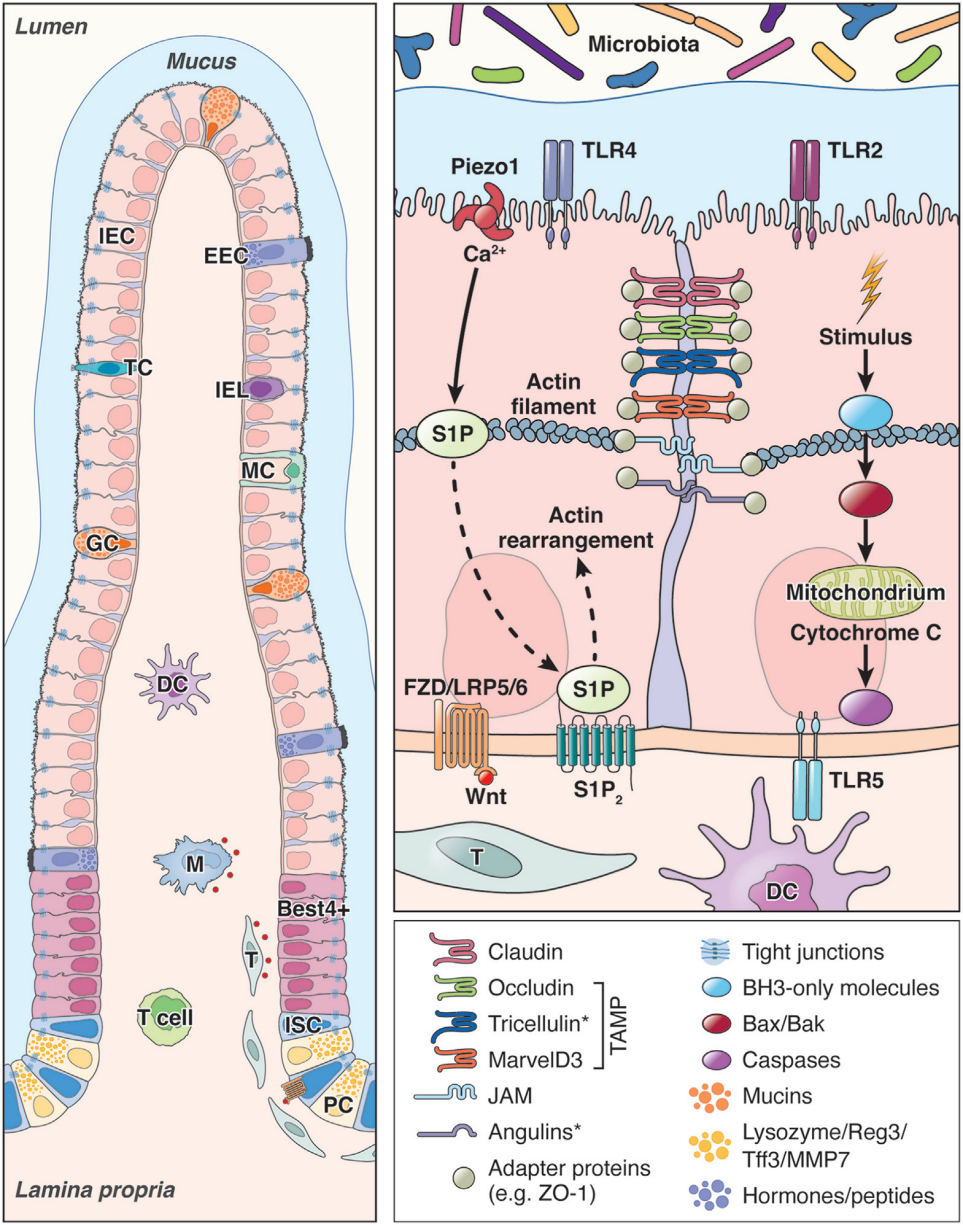
**Schematic representation of the small intestinal epithelium with the most important epithelial cell types, their cell-cell contacts, and their equipment to interact with immune cells and the microbiota.** DC, dendritic cell; EEC, enteroendocrine cell; IEL, intraepithelial lymphocyte; M, macrophage; MC, M-cell; T, trophocyte or telocyte; Tcell, thymocyte; TC, tuft cell. ∗Located at tricellular contacts.

The single-layered intestinal epithelium forms a protective barrier against the contents of the gut lumen with the help of specialized epithelial cells and a rigorous regeneration cascade. Intestinal stem cells (ISCs), located at the bottom of the crypts in the small and large intestine, serve as the foundation for this regeneration process. In 1974, Cheng and Leblond^6^ identified the so-called crypt-base columnar cells, which were later categorized as Lgr5-expressing (Lgr5+) multipotent stem cells.^7^ The stem cell niche is defined by a specific set of molecules, which are secreted by specialized cells in the crypt region. Among these molecules, Wnt molecules play a crucial role as signal molecules and have been reviewed extensively elsewhere.^8^ The canonical Wnt-β-catenin signaling pathway is vital for regulating the proliferation and differentiation of ISCs, ensuring the constant renewal of the intestinal lining. This pathway maintains intestinal homeostasis by controlling the balance between stem cell self-renewal and their maturation into specialized cell types.^9^ The primary sources of Wnt molecules are telocytes^10^ and trophocytes,^11^ which are located along the crypt-villus axis (Figure 1). Furthermore, there are interesting data on the role of T helper cells in stem cell modulation. Single-cell RNA Seq data revealed that stem cells express *Cd74*, encoding an invariant chain of the MHCII complex. Lgr5+ ISCs were found to interact with naive Th cells through presentation of ovalbumin via MHCII.^12^ Another publication by Cosin-Roger et al^13^ showed that the coculture of macrophages and Caco-2 cells led to the expression of Wnt ligands by polarized M2 macrophages and thereby significantly increased the nuclear accumulation of β-catenin and the expression of Lgr5 and c-Myc in the Caco-2 cells, speculating for the potential for stem cell proliferation. Interestingly, blocking CSF1R, the receptor for CSF1 and interleukin (IL)34, which caused depletion of dendritic cells in the lamina propria, led to disrupted Paneth cell (PC) differentiation and decreased the quantity of Lgr5+ ISCs.^14^ In summary, the crypt niche and cytokines from surrounding cells either promote or inhibit cell division, thereby influencing the differentiation of stem cells into mature intestinal epithelial cells (IECs).

### Differentiated IECs and Mucosal Immunology

The intestinal epithelium consists of 4 distinct cell types categorized into 2 main groups: secretory epithelial cells, such as PCs, goblet cells (GCs), and enteroendocrine cells; and absorptive epithelial cells, represented by enterocytes.^15^ The physical barrier formed by the epithelium serves as the primary protective barrier against the external environment. Specialized epithelial cells, including GCs, produce a mucus layer that progressively thickens from the small intestine to the large intestine. This layer is composed of glycoproteins called mucins and acts as a physical and chemical barrier, protecting the epithelium from pathogen invasion.^16^ Mucins are divided into 2 categories: transmembrane mucins that construct the glycocalyx^17^; and gel-forming mucins.^18^ The former are attached to the epithelial cells, whereas the latter are secreted by GCs and are released into the lumen. Mucins form a single mucus layer in the small intestine and a double layer in the colon, which efficiently guards the epithelial surface against bacterial invasion, because it was shown to be devoid of bacteria.^19^ GCs are situated along the crypt-villus axis and recent single-cell transcriptomics have identified numerous GC variants along the intestinal tract.^20^ A subpopulation known as intercrypt GCs represents differentiated GCs in the colon that are localized to the surface epithelium between crypts.^21^ In contrast to intercrypt GCs, crypt-resident GCs in the colon were identified to express the specific marker Mucin (MUC)5B,^20^ whereas sentinel GCs respond to toll-like receptor (TLR)2/1, TLR4, and TLR5 ligands by activating the Nlrp6 inflammasome, resulting in the release of MUC2.^22^ MUC2, which is important for gut homeostasis and tolerance, is one of the best-characterized mucins.^23^ Another cell type with secretory functions is PCs, which are typically located at the bottom of the crypt between the stem cells. A special signaling environment ensures the positioning of the PCs between the stem cells and prevents the PCs from moving upward along the villus to the tip.^24^^,^^25^ PCs have a significant defense arsenal, which includes various antimicrobial peptides stored in granules and can be released after stimulation. Alpha-defensins sourced from PCs can rupture the bacterial cell wall by forming pores or immobilize bacteria by forming a trap net.^26^ C-type lysozyme is a frequent constituent of PC granules and, like defensins, has the ability to break down bacterial cell walls. In contrast to mice, PCs can be found in the human cecum and ascending colon, and PC metaplasia in these regions can be found in IBD pathology.^27^ A study by Yu et al^28^ demonstrated that when lysozyme C-1 is genetically eliminated in mice, the immune response to bacterial molecular patterns in the colon is impaired, leading to an increase in the growth of lysozyme-sensitive mucolytic bacteria, such as *Ruminococcus gnavus*, a pathobiont linked to CD. The transfer of *R gnavus* into Lyz1-deficient mice triggered a type-2 immune response, with IL25 expressing tuft cells stimulating innate lymphoid cell (ILC)2 to secrete IL13. In contrast, processed *R gnavus* in lysozyme-proficient mice induced proinflammatory responses. Thus, PC lysozyme maintains a balance between anti-inflammatory and proinflammatory reactions in the gut.^28^ In addition to defensins and lysozyme, PCs secrete Reg3-alpha (human)/Reg3-gamma (murine), which binds to the peptidoglycan chains of the cell wall of gram-positive bacteria. Moreover, there are several other molecules produced or released by PCs that can act against microbes or their products, such as phospholipase A2, IgA, and MMP7, to name a few.^29^ Enteroendocrine cells are distributed throughout the gastrointestinal tract and are classified into various subgroups based on their location and shape.^30^ They primarily secrete hormones and peptides that affect adjacent cells. A study by van Es et al^31^ has demonstrated that enteroendocrine cells can act as alternative niche cells and ensure stem cell maintenance after PC ablation. M cells, residing within gut-associated lymphoid tissues, play a significant role in mucosal immune regulation. These cells collect antimicrobial components from the intestinal lumen and hand them over to macrophages or present them directly to dendritic cells residing in the lymphoid follicle.^32^ Tuft cells are named after their strong apical microvilli apparatus and are known for their ability of recognizing intestinal helminths. They can activate ILC2 in the lamina propria by the secretion of alarmins, such as IL25.^33^ Bestrophin-4-positive (Best4+) cells are a newly identified cell type found in the human small and large intestine. Studies using diffusion pseudotime, a method for estimating the temporal sequence of differentiating cells in single-cell RNA sequencing, have shown that these cells share similarities with secretory progenitors or maturing cells. Notably, Best4+ cells are absent from crypts in the small intestine.^20^^,^^34^ These cells can be characterized as mature absorptive cells with potential functions that include the recognition and regulation of luminal pH, the conversion of electrolytes, and the secretion of antimicrobial peptides.^35^ Differential IECs possess various mechanisms to maintain homeostasis. Nonetheless, a constant proliferation of new IECs also elevates the necessity to discard aging IECs at the villus tip. This process must also be strictly regulated.

### The Role of Tight Junction Proteins to Maintain Intestinal Barrier Function

In the intestinal barrier, transport properties heavily rely on paracellular characteristics apart from transcellular transport through channels, carriers, and transporters. These are determined and regulated by tight junction (TJ). TJs are the most apical located cell-cell contacts in epithelia. They seal the paracellular cleft, maintain epithelial polarity, and determine selective paracellular permeability properties in the epithelium. These functions are achieved by specific combinations of TJ proteins, which include 4 different families of transmembrane protein.

The family of claudins, which in mammals contains 27 members,^36^ is the protein family with the main influence on functionality of the TJ. Although most of these tetraspan proteins have barrier-sealing properties there are also several known to form paracellular channels allowing size- and charge-selective permeability. Among the claudins expressed within the intestine, claudin-2, -15, and -10b are forming cation-selective channels.^37^, ^38^, ^39^ Besides their cation-channel properties, claudin-2 and -15 also form paracellular channels that are permeable for water.^40^^,^^41^ In addition to homomerically formed channels, heteromeric channels can be formed, which is so far described in the kidney for claudin-16 and -19 that form cation-selective channels only when combined.^42^ Although not yet described for intestinal claudins, the formation of specific functionality caused by heteromeric interaction may be assumed, because the specific expression, localization, and interaction patterns of claudins determine the functional features of tissues and organs. Claudin-23, for example is not limited to luminal IECs, where it tightens the barrier, but also can recruit and interact with claudin-3 and -4 to TJs.^43^ Claudins may not only reside at TJs, because some claudins, such as claudin-1, -3, -4, -5, and -7, have also been shown to localize to the plasma membrane of enterocytes, suggesting other noncanonical functions.^44^ For instance, claudin-7 plays a critical role in regulation of crypt stem cell function^45^ and is maintaining Wnt and Notch signaling pathways in the colonic epithelium.^46^

The family of TJ-associated marvel (myelin and lymphocyte and related proteins for vesicle trafficking and membrane link) proteins (TAMP) comprises the tetraspan membrane proteins occludin, tricellulin, and marvelD3. These proteins have regulatory functions but also influence barrier function (Figure 1). Occludin, as first discovered TJ protein,^47^ is a protein with variable barrier properties depending on the model studied. Although it does influence transepithelial resistance and ion permeability in certain models,^48^^,^^49^ it has mainly regulatory functions in others,^50^^,^^51^ such as regulating epithelial apoptosis and proliferation, or being involved in specialized epithelial structure organization.^52^ Although its influence on macromolecule permeability seems to differ as well among in vitro studies,^53^, ^54^, ^55^ knockdown in mice by siRNA leads to increased macromolecule permeability.^56^ For knockout mice, permeability for inulin was reported to be increased in the colon,^57^ supporting that occludin is involved in paracellular macromolecule permeability, probably together with another TAMP, tricellulin, because loss of occludin leads to localization shifts of tricellulin.^58^ Tricellulin usually is predominantly located within tricellular contacts, the meeting points of at least 3 cells and their TJs.^59^ These converge and extend laterally, forming tricellular TJs (tTJ). Here, tricellulin has tightening functions mainly affecting permeability for macromolecules,^59^, ^60^, ^61^ whereas in tight epithelia, it may also influence paracellular ion and water permeability.^60^^,^^62^

For the third TAMP marvelD3, mainly regulatory functions have been observed including TJ stabilization, cell behavior and survival, and development.^63^, ^64^, ^65^, ^66^, ^67^

The family of angulins has 3 known members: angulin-1, also referred to as lipolysis stimulated lipoprotein receptor or as immunoglobulin like domain containing receptor (ILDR)^3^^,^^68^^,^^69^ and ILDR1 and ILDR2.^70^ These proteins are single-span and often have tricellular localization. They comprise the basic structure of tTJ and ensure the correct tricellular localization of tricellulin.^70^^,^^71^ Thus, they indirectly and directly impact tricellular permeability of macromolecules and water.^72^^,^^73^

The junctional adhesion molecule (JAM) family includes single-span proteins, which fulfill adhesive functions, act as interaction partners with leukocytes, and regulate epithelial and endothelial barrier formation.^74^ Moreover, they play important roles in hemostasis, angiogenesis, hematopoiesis, germ cell development, and the development of the central and peripheral nervous system.^75^^,^^76^ Besides these regulatory functions, JAM-A is also an essential adhesion factor, because deletion of JAM-A^77^ in mice leads to compromised intestinal barrier function, affecting ion and also macromolecule permeabilities but not leading to enhanced translocation of bacteria. The knockout also increased proliferation and induced alteration in expression of other TJ proteins.

Apart from the membrane-passing TJ proteins, a variety of TJ-associated cytoplasmic scaffolding proteins^77^ link the TJ to the cytoskeleton and have many other, mainly regulatory functions. These functions allow the barrier to respond to changes in environmental conditions.^78^^,^^79^ The most familiar TJ marker is zonula occludens 1, which has numerous crucial functions.^52^

TJ proteins, especially claudins, exhibit segment-specific expression and localization, regulating localized barrier and permeability properties based on the respective tissue’s functionalities. In the intestine, the expression of claudins varies in the proximal and distal segments,^80^^,^^81^ as indicated in Table 1. In the leakier proximal segments, which are characterized by high ion and water permeability, the channel-forming claudins, specifically claudin-2 and -15, can be found. Claudin-2, for instance, is located in the surface epithelium of the duodenum, jejunum, and ileum. For claudin-15, research indicated that it mediates sodium back-leak in the small intestine, which is crucial for sugar absorption through the sodium glucose transporter 1.^82^

**Table 1:** Intestinal TJ Protein Expression, Localization, and Effects in IBD

	Small intestine	Colon	UC	CD
Claudins				
Claudin-1 (TJ+bl)^83^	Crypt+villus^83^	Crypt^84^^,^^83^^,^^85^	↑ / shift to bl^86^^,^^87^^,^^88^ = ^89^	↑ / shift to bl^90^ = ^91^^,^^89^^,^^84^
Claudin-2 (TJ)^91^^,^^83^	Crypt base^92^	Crypt base^91^^,^^83^^,^^92^	↑ ^81^^,^^93^^,^^86^	↑ ^91^^,^^90^ ↓ ^81^
Claudin-3 (TJ+bl)^91^^,^^83^	Crypt: low Villus: high^94^^,^^95^	Crypt base: low Crypt surface: high^92^	↓ ^96^^,^^97^ = ^81^^,^^89^	↓ / shift to endosomes^81^^,^^91^^,^^90^
Claudin-4 (TJ+bl)^83^	Villus apex^92^^,^^98^	Crypt surface^92^	↓ ^89^ = ^81^	= ^81^^,^^91^^,^^90^
Claudin-5 (TJ+bl)^83^	Crypt+villus^92^	Crypt^91^ Crypt base^92^		↓ / shift to bl^91^
Claudin-7 (TJ+bl)^83^	Crypt: low Villus: high^95^	Crypt^83^^,^^99^	↓ ^89^	= ^91^^,^^90^
Claudin-8 (TJ)^91^^,^^83^	Crypt+villus^99^	Midcrypt^83^		↓ / shift to endosomes^91^^,^^88^
Claudin-10 (TJ)^83^	Crypt: high Villus: low^83^	Crypt base: high Crypt surface: low^83^		
Claudin-12 (TJ)^83^	Crypt+villus^99^	Crypt^99^	= ^81^	↓ ^81^^,^^88^
Claudin-13^a^ (TJ+bl)^99^	NA	Crypt^99^		
Claudin-15 (TJ)^83^	Crypt: high Villus: low^83^	Crypt base^99^	↓ ^100^	
Claudin-18 (TJ)^83^	ND	ND	↑ ^88^^,^^101^	
Claudin-23 (TJ)^43^	ND	Crypt base: low Crypt surface: high^43^		
TAMPs				
Occludin (TJ)^102^	Crypt+villus^102^	Crypt^102^	↓ ^93^	↓ ^91^
Tricellulin (tTJ)^59^	ND	Crypt^70^^,^^103^	↓ ^103^	= / shift to surface^103^
MarvelD3 (TJ)^63^	ND	Crypt^104^	↑ ^104^	= ^104^
Angulins				
Angulin-1 / LSR (tTJ)^68^^,^^69^	Crypt+villus^70^ + bl^105^	Crypt base^70^	= ^106^^,^^107^	↓ ^106^^,^^107^
Angulin-2 / ILDR-1 (tTJ)^70^	ND	Crypt^70^	= ^106^^,^^107^	= ^106^^,^^107^
Angulin-3 / ILDR-2 (tTJ)^70^	ND	ND	= ^106^^,^^107^	= ^106^^,^^107^
JAMs				
JAM-A (TJ)^108^^,^^109^	Crypt+villus^108^	ND	↓ ^84^^,^^110^	↓ ^84^^,^^110^
JAM4 (TJ)^111^^,^^112^	ND	ND		

NOTE: Localizations were mainly analyzed in rodent tissue. Expression analysis in IBD contains mRNA and/or protein data.

bl, basolateral; CD, Crohn’s disease; IBD, inflammatory bowel disease; ILDR, immunoglobulin-like domain containing receptor; LSR, lipolysis stimulated lipoprotein receptor; NA, not applicable; ND, exact localization not determined; TAMP, tight junction-associated marvel proteins; TJ, tight junction; tTJ, tricellular TJ; UC, ulcerative colitis; ↑, upregulated; ↓, downregulated; =, unaffected.

a Rodent-specific claudin.

In general, the tightness in the distal segments stabilizes the possibility of forming gradients between the luminal and serosal side. This is achieved through a tight and nearly impermeable TJ, predominantly comprised of tightening claudins, including claudin-1, -3, -4, -5, -8, and -23. In this location, channel-forming claudins, specifically claudin-2 and -15, are also expressed but only in the depths of the crypts. Some TJ proteins serve very specific functions in the intestine. For instance, claudin-12, along with claudin-2, plays an essential role in vitamin D–regulated Ca^2+^-uptake,^113^ and is predominantly found in the surface epithelium of the jejunum, with decreasing levels observed toward the colon.

### Controlling Intestinal Epithelial Cell Death to Maintain Epithelial Homeostasis

IECs originate at the crypt base, differentiate, and position themselves along the villus until they are replaced by new IECs every 3–5 days.^114^ To cope with the continuous supply of new differentiated IECs, the turnover of these cells at the tip of the villus is required but at the same time must be strictly regulated. *Anoikis*, the ancient Greek word for homelessness, refers to a form of cell death at the villus tip caused by the detachment of cells from the extracellular matrix or from neighboring cells.^115^ The detachment of IECs from the extracellular matrix, also known as cell shedding or cell extrusion, results in anoikis, preventing uncontrolled breakdown of the epithelial barrier and potential inflammatory responses. Shedding is a process typically seen at the top of the villus where the pressure from crowding IECs is greatest.^116^ Physiological shedding is sensed by the mechanosensitive ion channel Piezo1, which can signal either crowding or stretching, depending on the localization within the cell.^117^ Piezo1 in epithelial cells is activated on mechanical force, which leads to the release of sphingolipid-1-phosphate (S1P) in the extruding cell and which is exported to neighboring cells.^116^ S1P binds to S1P2 (S1P2) in the neighboring cells, which leads to activation of Rho and triggers an actin/myosin ring formation in the neighboring cell to force the dying cell to be squeezed out of the epithelial layer (Figure 1).^118^ Knocking down Piezo1 in zebrafish prevented cell shedding and led to the accumulation of epithelial cells.^116^ Disengagement of integrins from the extracellular matrix leads to the release of Bmf, a proapoptotic BH3-only protein that inactivates the antiapoptotic functions of Bcl-2. This enables the oligomer formation of Bax and Bad, which in turn trigger the perforation of the mitochondrial membrane and the initiation of apoptosis,^119^^,^^120^ through the release of cytochrome *c*.^121^ The presence of cytochrome c in the cytoplasm and the binding to APAF-1 activates the procaspase-9. This in turn leads to the activation and cleavage of caspases, such as caspase-3 or caspase-7, which destroy important structural proteins and activate enzymes that ultimately lead to cell rupture.^122^

Extrinsic signals can also cause apoptosis of IECs and disrupt the gut barrier. In this case the binding of an extracellular ligand, such as FasL/CD95L or tumor necrosis factor (TNF)-α, to specific receptors or the release of cytolytic granules by CTLs and natural killer cells, which can then directly activate caspases, trigger apoptotic cell death. In receptor-mediated apoptosis activation, binding of the ligand to the receptor recruits the adapter molecule FADD (Fas-associated death domain protein) to the receptor. This leads to the formation of an intracellular death-inducing signaling complex. Caspase-8 is then recruited to the death-inducing signaling complex and cleaved and activates downstream effector caspases.^123^ To maintain gut homeostasis, the cell must die without leading to an inflammatory reaction. This is achieved by the release of find-me signals, such as S1P or ATP, followed by eat-me signals, such as phosphatidylserine changes in the charge or glycosylation patterns on the cell surface. Finally this leads to degradation of the cell and uptake of the remains by phagocytes.^124^

Another form of regulated cell death in the epithelium under physiological, but also pathologic, conditions is autophagy. Autophagy occurs when cells are under metabolic stress (eg, endoplasmic reticulum [ER] stress, oxidative stress, or pathogen infection).^125^ During autophagy epithelial cells engage in a process of intracellular breakdown and recycling, wherein the cellular machinery breaks down and recycles its own components within the confines of the cell. The ULK1 complex initiates autophagy.^126^ Together with the PtdIns3K complex, it is part of the autophagosome machinery located in the rough ER. By working with other complexes, the phagophore is created to trap autophagic targets. Subsequently, these targets are degraded and used through lysosomal fusion.^127^ Autophagy is considered a vital mechanism for IECs to maintain homeostasis. The most significant anomaly in the genetic depletion of autophagy genes was found in PC defects, resulting in PC granule content secretion impairment^128^^,^^129^ or in changes of the ultrastructural morphology of the PCs.^130^ In cell culture experiments, it was shown that starvation-induced autophagy in Caco-2 cells increased the presence of the pore-forming TJ protein claudin-2 in the cytoplasm and lysosomes, supported the stabilization of the localization of the protein occludin at the TJs, and reduced the paracellular permeability of small urea molecules.^131^^,^^132^ Autophagy was also shown to affect the stem cell niche and the formation of facultative stem cells. On injury, facultative ISCs, such as secretory progenitor cells, can dedifferentiate into a stem cell state and proliferate to replenish the lost or damaged cells. It was shown that cells with high levels of autophagic vesicles show plasticity in vitro and are protected from injury in vivo.^133^^,^^134^

Other forms of inflammatory cell death can occur in IECs; however, in this review we focus on the 3 main types of cell death specific to IECS: apoptosis, autophagy, and anoikis.

### Dysfunction of the Intestinal Barrier in IBD

In the last 50 years, there has been an increase in the incidence of IBD, also in the elderly and in developing countries.^1^^,^^135^ Despite significant research efforts and the development of various biologic therapies aimed at targeting specific inflammatory pathways, a definitive cure remains elusive. Patients with IBD suffer from recurrent inflammation of the intestinal tissue, which in the case of CD typically targets the terminal ileum but has the capability to affect any region of the gastrointestinal tract, whereas in UC it is limited to the colon. Although in UC only the mucosa is affected, in CD inflammation may span across all tissue layers of the gut. Both diseases enormously reduce the quality of life of those affected and are often accompanied by further complications that require surgical treatment.^136^ In the following section, focus is laid on dysregulations in IECs or surrounding cells and factors that may lead to the development of IBD.

### Disturbances in the Intestinal Stem Cell Niche during IBD

As previously discussed, Lgr5+ ISCs maintain the epithelial cell barrier and thus control the generation of new IECs.^114^ In the event of injury or inflammation and subsequent destruction of the Lgr5+ ISCs, reserve ISCs (also known as quiescent or dormant stem cells) can take over the generation of new IECs.^137^^,^^138^ In addition, cell lineage progenitors have been shown to dedifferentiate and form new IECs during inflammation and tissue destruction. Indeed, Lgr5+ stem cells are dispensable for epithelial regeneration after experimental colitis.^139^ For example, secretory progenitors expressing ATOH1 can exhibit ISC properties after injury. Using the experimental dextran sulfate sodium (DSS) colitis model, Ishibashi et al^140^ demonstrated that ATOH1+ IEC-derived cells are involved in the formation of wound-associated tissue. They could also show that a cocktail of TNF-α, IL1β, and flagellin was able to induce the formation of ATOH1+ IEC-derived cells.^140^ The mesenchymal niche also plays an important role in epithelial regeneration. Knockout models have shown that myofibroblasts are the source of stromal Rspo3, which is essential for the regeneration and differentiation of Axin2- crypt cells after destruction of Lgr5+ and Axin2+ cells following colonic inflammation.^141^ In addition, BMP4 has been shown to be activated via ID3 in ISCs, thereby attenuating DSS-induced inflammation in mice. BMP4 is mainly secreted by intravillus and intervillus mesenchymal cells and is responsible for the differentiation gradient of the crypt/villus axis.^142^ Olfm4, a robust marker for ISCs, was shown to disappear from the intestinal epithelium after Foxl1 deletion.^143^ A similar mechanism was shown for Gli1+ mesenchymal cells. After genetic blockade of Wnt secretion in Gli1+ cells, protein expression of Olfm4 in IECS was lost. During recovery from DSS colitis, an increase in Gli1+ cells was observed, which contributed to the restoration of tissue integrity.^144^ A recent single-cell RNA-Seq study identified 12 distinct clusters of mesenchymal cells in patients with UC and 13 cell clusters were identified in DSS-treated mice (an additional population of pericytes). Surprisingly, the most important subset-specific marker pairs were also found in the mouse.^145^ Studies like these will help to develop future generations of Cre-expressing reporter mouse lines to study the functions of mesenchymal subtypes in vivo. Stromal cells can also help to regenerate the inflamed epithelium indirectly through communication with immune cells. For instance, loss of Ihh in IECs led to migration of immune cells through the release of CXCL12 exclusively by fibroblasts.^146^ Oncostatin M is a cytokine produced by stromal cells. Inflammation-associated fibroblasts respond to the released Oncostatin M by expressing the receptor and produce proinflammatory molecules, which in turn attract T cells or neutrophil granulocytes that cause an inflammatory response.^147^ In addition to macrophages, myofibroblasts can also produce TNF-α, one of the best-known cytokines in IBD, creating a proinflammatory milieu. Current treatment options include anti-TNF antibodies, such as infliximab, in patients with CD. A study by Di Sabatino et al^148^ showed that myofibroblasts from patients with CD expressed higher levels of TNF-α than control subjects and treatment with infliximab, among others, resulted in increased TIMP-1 production and migration of the myofibroblasts, which may lead to improved wound healing. A therapeutic strategy based on these findings is the mesenchymal stromal cell (MSC) therapy. The preferred local application of MSCs could lead to an alleviation of DSS colitis in mice.^149^ Exosomes from MSCs administered during DSS colitis in mice resulted in decreased expression of inflammatory cytokines in colonic macrophages, which was required for suppression of inflammatory responses.^150^ The therapeutic potential of MSC-based therapy was reviewed in detail elsewhere.^151^

### Intestinal Epithelial Cell Crosstalk to the Microbiome during Inflammation

Epithelial cells and their communication with immune cells and the microbiota are of particular importance for the development of IBD. Bacteria can interact with the epithelium directly (eg, via cell wall components called PAMPs). Additionally, bacteria can indirectly act on the epithelium via their metabolites. Specific innate receptors on IECs recognize these components and metabolites. TLRs are one of the most important microbial pattern recognition receptors. While under homeostatic conditions in mice, TLR2 and TLR4 have been shown to be expressed rather weakly in the small intestinal epithelium but are higher expressed in IECs of the proximal and distal colon.^152^ The expression of TLR5 was found in PCs in the small intestine and in the proximal colon, but not in the distal colon (Figure 1). TLR7 and TLR9 were not detected in the epithelium under homeostatic conditions and expression was restricted to the lamina propria.^152^ After activation of TLRs, for example, through lipopolysaccharides or other PAMPs, the signaling cascade continues through the 2 major adaptor proteins MyD88 and TRIF, ultimately leading to the release of proinflammatory cytokines, such as TNF-α, IL6, IL8, or CCL20.^153^ TLR4-deficient mice showed more intestinal inflammation when treated with DSS compared with control subjects.^154^ However, infection of TLR4-deficient mice with *Citrobacter rodentium* showed a protective effect and did not exacerbate intestinal inflammation.^155^ This suggests a balancing role of TRL4 in relation to inflammation, infection, or wound healing, which is triggered depending on the type of stimulus. In humans, TLR4 deficiency has been linked to the development of IBD.^156^ TLRs are only 1 class of pattern recognition receptors on IECs. One of the best studied risk genes for CD is NOD2, which codes for an intracellular receptor that recognizes bacterial peptidoglycans. Several gene variants have been identified to be associated with CD.^157^ NOD2 signals via nuclear factor-κB and initiates the release of proinflammatory cytokines but also of regenerative factors. Alterations in NOD2 expression or function may therefore lead to a dysregulation of microbial handling and thus to excessive inflammation. It was shown that ISCs in particular, in addition to PCs, express NOD2 and that NOD2 in these cells contributes to stem cell survival and the prevention of cell death.^158^ Another example of epithelial dysfunctions influencing the microbiota in patients with IBD is the mucin MUC2. MUC2 is produced by GCs and patients suffering from CD show reduced levels of MUC2.^159^ Furthermore, patients with UC show reduced O-glycosylation of MUC2 and an increase in *Escherichia coli*, postulating to influence the activity of the NFKB signaling pathway in the mucosal lining.^160^ Likewise, other GC-specific factors that are released during inflammation are dysregulated in patients with IBD. Activation of TLR2 leads to the secretion of trefoil factor (TFF) 3, which is important for mucosal defense and is secreted by GCs. Mice with genetic ablation of TLR2 or TFF3 show increased epithelial cell death and intestinal inflammation.^161^ TFF3 is upregulated at the site of mucosal damage and known for its protective function.^162^ TFF3 serum levels are increased in patients with UC and administration of TFF3 dimer significantly ameliorated the severity of chemically induced colitis in mice.^163^^,^^164^ Functional studies showed that WFDC2, an antibacterial protein, is highly expressed in GCs of the colon under homeostatic conditions, and is significantly downregulated in inflamed tissues of patients with UC.^165^

It is assumed that the gut microbiota of patients with IBD has a lower biodiversity and taxonomic and functional shifts, known as dysbiosis.^166^^,^^167^ Whether these changes are the cause or the consequence of inflammation in patients with IBD cannot be answered so easily. The microbiota associated with IBD is characterized by a relative increase in the abundance of Bacteroidetes and Proteobacteria, along with a concomitant decrease in Firmicutes, compared with healthy control subjects.^168^ *Lactobacillus rhamnosus GG* is one of the Firmicutes that has been administered as probiotics to patients with CD to maintain remission.^169^ Several cell culture studies have addressed the protective properties of different *L rhamnosus* strains. Accordingly, it was shown that *L rhamnosus GG* produce soluble proteins that attenuate the decrease in transepithelial resistance caused by hydrogen peroxide and the increase in inulin permeability in Caco-2 cells.^170^ In a study by Zheng et al,^171^ the damage to Caco-2 cells by lipopolysaccharides could be attenuated via the inhibition of the TLR4/nuclear factor-κB signaling pathway by the administration of *L rhamnosus CY12*. Another example of microbial changes in patients with CD is a reduced number of *Roseburia intestinalis*. In 2 different mouse models, DSS- and TNBS-induced colitis, the supernatant of *R intestinalis* was shown to ameliorate colitis by reducing the number of inflammatory macrophages and Th17 cells in the colon and downregulating the expression of IL6 and STAT3.^172^ In patients with CD and patients with UC a reduction in *Faecalibacterium prausnitzii* could be observed. In a mouse study, *F prausnitzii* was shown to upregulate the expression of Dact3, a gene linked to the Wnt/JNK pathway, which is important for the maintenance of epithelial cell homoeostasis.^173^

Bacterial metabolites, such as short-chain fatty acids also have a major influence on the regeneration of epithelial cell tissue. Kaiko et al^174^ showed that the architecture of the colon shields colonic stem cells from the impact of butyrate. Experiments with DSS showed that destruction of the epithelium allows butyrate to reach the stem cells, leading to reduced proliferation and increased ulcer formation. Treatment with metronidazole, which eliminated butyrate-producing bacteria, led to a reduction in ulcer formation. The aryl hydrocarbon receptor (AhR) has, among others, indole-based metabolites as ligands, which are produced by bacteria of the intestinal microbial flora. In mice, these nuclear receptors are mainly expressed by Lgr5+ stem cells. IEC-specific AhR knockout mice showed increased microbial penetration capacity and differences in the expression of GCs and enterocyte markers after infection with *C rodentium*, suggesting a deficit in differentiation of these cell types.^175^ Immune cells located beneath the intestinal epithelium in the lamina propria react to a disturbed intestinal flora or a barrier defect. ILCs and dendritic cells and natural killer cells can produce IL22, a cytokine that stimulates the production of antimicrobial peptides, especially in IECs.^176^ Intraepithelial lymphocytes are scattered along the epithelium and colocalize with IECs. A recent study uncovered several subtypes of intraepithelial lymphocytes in inflamed areas of the intestinal mucosa of patients with CD, and there is evidence of the role of intraepithelial lymphocytes during intestinal inflammation ranging from proinflammatory to anti-inflammatory.^177^^,^^178^ Furthermore, activated AhR receptors directly regulate the expression of barrier-forming claudin-3 in IECs.^179^ In a mouse study using DSS-induced intestinal inflammation it was shown that the activation of AhR by FICZ, a high-affinity ligand for AhR, led to reduced severity of mucosal inflammation in mice compared with control subjects. The FICZ-treated group showed a prevented reduction in TJ protein expression, such as claudin-1 or CYP1A1, suggesting an ameliorated disease outcome by AhR activation.^180^ The constant interaction of epithelial cells with the microbiota and signal transduction to immune cells, and feedback from immune cells to the epithelium, provides the basis for the maintenance of the intestinal barrier.

### Tight Junction Regulation during Intestinal Inflammation

During inflammatory processes as in IBD, all intestinal barriers are impacted resulting in microbial imbalances, immune cell activation, and compromised epithelial barrier properties that are markedly caused by alterations in the TJ. Already early observations of the ultrastructural TJ network indicated massive changes in its composition, because the continuous, linear strand pattern of TJs, which is typical for tight epithelia, is altered to a particle-type appearance with strand breaks and loss of continuity in IBD, accompanied with as reduction of horizontally oriented strands.^93^^,^^91^ Meanwhile on a molecular level, expression and localization changes have been described, which are either common for both main entities of IBD or are specific for one or the other.

A common TJ protein upregulated during inflammation and infectious conditions is claudin-2. Its presence results in an increase in paracellular cation and water permeability, leading to one of the primary symptoms of acute IBD, leak flux diarrhea. Under physiological conditions, the nearly impermeable TJs of the large intestine allow the formation and maintenance of gradients, as transcellularly absorbed ions cannot flow back via the paracellular cleft. For example, in the colon because of the electrogenic absorption of Na^+^ via the epithelial sodium channel (ENaC) gradients with a negatively charged lumen are built. The introduction of the cation- and water-permeable channel claudin-2 into the otherwise tight TJ enables backflow of cations in the lumen, which is driven by the initially present charge and ion gradient. This flow of cations into the lumen and the resulting osmotic imbalance force water to follow, either via claudin-2, via aquaporins, or via larger leaks, and leads to one of the main symptoms of acute IBD, leak flux diarrhea.^181^

Although long believed to be a massive disturbance worsening the disease, it meanwhile has been shown that this water-flow induced by claudin-2 upregulation has more ameliorating effects, because in colitis models using claudin-2 knockout mice disease activity was higher.^182^^,^^183^ The function of this leak flux seems to be rinsing the intestinal epithelium to wash away pathogens or inflammation-inducing factors, similar to the mechanism of “enteric tears.”^184^ However, claudin-2 upregulation promotes progression of immune-mediated experimental colitis underlining that claudin-2 has complex functions in mucosal (patho)physiology.^185^ Proinflammatory cytokines, such as TNF-α^91^ and IL13,^93^ are linked to the observed claudin-2 increase. The other important channel-forming intestinal claudin, claudin-15, has been described as downregulated in UC.^100^

For tightening claudins, mainly downregulation has been observed in IBD (Table 1), which leads to weaker barrier properties in general and thus further breakdown of physiological gradients as mentioned previously. For example, claudin-4 and -7 are downregulated in UC,^89^ and claudin-5 and -8 in CD.^91^ Of note, several cytokines play a role in these changes. For instance, TNF-α causes the dislocation of claudin-5 and -8 from the TJ to sub-TJ membrane compartments and into endosomes.^91^ That not only the expression but also the localizations of the respective claudins are of relevance can be seen on the example of the “claudin-1 paradox.” Claudin-1 was reported to be significantly reduced in epithelial cells of patients with IBD^84^ but other studies showed its upregulation in UC and Crohn’s colitis.^86^^,^^87^ Although this seems to contradict a weaker and impaired barrier, the higher claudin-1 levels occur mainly outside the TJ areas^86^ where it is less relevant for the actual TJ barrier function. Such an upregulation can be interpreted as the onset of a counterreaction and increased claudin-1 levels have further been shown to play a complex role in the development, phenotype, and prognosis of colitis-associated cancer and are also used as a biomarker in this context.^186^

Changes in general permeability properties covering ions, water, and large solutes may be explained by the orchestra of the claudin expression changes and the subsequent serious impairments of the TJ strand network leading to strand discontinuities and breaks. However, increased luminal macromolecule uptake is also observed under less severe conditions and can also be used for the prediction of relapse in apparent remission.^187^ On the paracellular level, macromolecule passage may not be linked to the size-restrictive claudins but is more likely to changes in occludin, tricellulin, and JAM.

Among the family of TAMP, occludin downregulation has been reported for CD^153^ and for UC.^93^ The cytokines involved are interferon-γ and TNF-α^188^ and as described previously the reduction of occludin may contribute to increased macromolecule permeability.^54^^,^^56^ This could be either direct or indirect, because occludin reduction has also been shown to cause shifts in the localization of tricellulin from the tTJ to the bicellular TJ.^54^^,^^58^ In addition to such a shift in localization, tricellulin itself and thus the tTJ barrier are also affected in IBD, further influencing the barrier to macromolecule passage. In UC, tricellulin is downregulated.^103^ This downregulation can be achieved by IL13 and is accompanied by increased macromolecule passage. In CD, however, expression remained unaffected. Here, a shift in localization of tricellulin was observed,^103^ which was suggested to depend on angulin-1, for which expression is reduced in CD.^106^^,^^107^ Downregulation of angulin-1 is controlled by leptin-1, an adipokine that is secreted by creeping fat, one of the typical features of CD.^104^, ^189^, ^190^ The other angulins have been reported as unaffected in IBD.^106^^,^^107^

The third TAMP, marvelD3, is upregulated in UC, and the cytokine involved here is IL13.^191^ As for marvelD3, more regulatory functions have been described,^63^, ^64^, ^65^, ^66^, ^67^ but the actual role for the TJ barrier in IBD is still not clear. However, the upregulation was shown to have ameliorating effects on colitis severity in a mouse model.^191^ Also, for JAM-A regulatory roles in proliferation and intestinal homeostasis during inflammation are assumed but also important for the macromolecule barrier,^192^ especially because JAM-A is downregulated in UC and CD.^84^^,^^110^ In vitro, JAM-A-deficient cells undergo spontaneous cell stretching also leading to junction fracture, which is accompanied by actin disorganization; and actin polymerization is required for apical junction.^193^

TJ proteins are linked to the actomyosin cytoskeleton via scaffolding proteins.^77^ Important in this context are especially nonmuscle myosin IIA and myosin II regulatory light chain (MLC). Nonmuscle myosin IIA plays a relevant role in establishing the normal intestinal barrier and in protection against mucosal inflammation in vivo.^194^ Phosphorylation of MLC by MLC kinase regulates TJ proteins,^195^ driving intestinal TJ permeability triggered by exposure to cytokines, such as TNF-α,^196^ showing that cytokines do not only directly affect expression of TJ proteins as mentioned previously but also influence the TJ regulatory via the cytoskeleton. Knockout of MLC kinase and inhibition of its recruitment to the perijunctional actomyosin belt, prevents MLC phosphorylation, the subsequent occludin endocytosis,^197^ and barrier loss.^198^^,^^199^

### Intestinal Epithelial Stress and Cell Death in Intestinal Inflammation

Dysregulation of epithelial cell death is frequently linked to the emergence of intestinal inflammation. The shedding of aged epithelial cells at the villus tip is mainly carried out by the ion channel Piezo1. Surprisingly, Piezo1 has been shown to be upregulated in the epithelium of patients with CD, causing calcium influx, which in turn leads to mitochondrial dysfunction and activates NLRP3 inflammasome-mediated inflammation.^200^ Molecules important for cell shedding also include GTPases and GGTases. For example, mice with a genetic deletion of RhoA or RAC1 or the geranylgeranyltransferase Pggt1b (GGTase-1) in IECs exhibit cytoskeletal restructuring and abnormal cell shedding.^201^^,^^202^ Experiments with knockout mice lacking apoptosis genes in the intestinal epithelium have demonstrated that apoptosis is an important process to remove epithelial cells without disrupting the epithelium and the most important mediators for this are caspases. Interestingly, single knockout of caspase-3 or -7 or double knockouts of caspase-3 and -7 showed no morphologic changes in the intestinal architecture when compared with control subjects at steady state conditions.^203^^,^^204^ In contrast, caspase-8-deficient mice in the intestinal epithelium develop spontaneous ileitis (and depending on the microbial flora also colitis) and exhibit a lack of PCs and dysregulated antimicrobial functions.^205^ Another factor that can trigger cell death in IECs is cellular stress. Because PCs are also important for ISC survival by secreting Wnt and other growth factors to maintain stemness, it is no surprise that a knock-in inducing the polymorphism of the T300 A variant of the Atg16L1 gene, one of the main autophagy-related genes, in IECs led to disturbances in ISC maintenance with fewer Lgr5+ ISCs resulting in lower organoid formation.^206^ Studies by Cadwell et al^128^ have demonstrated that PCs are affected by Atg16L1 deficiency in showing abnormalities, such as disorganized granules and decreased granule numbers. Furthermore, lysozyme, typically confined within intact granules in PCs, was found to be diffusely distributed.^128^ Moreover, mice with a genetic ablation of ATG16L1 displayed structural and granule packaging abnormalities in their PCs on infection with murine norovirus.^130^ This study also revealed that the induction of colonic inflammation by DSS in these mice resulted in similar pathologic conditions as in patients with CD. These conditions included inflammation of the muscularis and associated mesenteric fat, and the development of subserosal fibrosis. In another study, deletion of Atg5, an essential component of the autophagy machinery, in IECs resulted in greater accumulation of mucin-filled granules compared with control subjects with a defect in granule exocytosis, which is caused by insufficient generation of reactive oxygen species.^207^ In addition, other secretory cells, such as GCs, also showed malfunctions when autophagy genes were deleted in mice. The use of Becn1F121A mice, in which autophagy is constitutively activated, led to reduced ER stress and excessive mucus production.^208^ Resilience against ER stress in IECs is an important mechanism to maintain intestinal homeostasis and epithelial cell function. Xbp1 and IRE1 are important factors of the ER stress response.^209^ Interestingly, genetic knockout of Xbp1 in IECs showed almost complete absence of PCs and hyperproliferation of IECs.^210^ Furthermore these mice showed an increase in IgA+ plasma cells^211^ and an expansion of Th17 cells,^212^ changes that can also be observed in patients with IBD.^213^, ^214^, ^215^ Mice with a genetic knockout of IRE1 showed a loss of GCs and disruption of the barrier function of the intestinal epithelium and an increased susceptibility to chemically induced colitis,^216^ underlining the key role of ER stress for gut homeostasis.

Previous data highlight a role for necroptosis in intestinal inflammation. The initial demonstration of the involvement of necroptosis in the development of intestinal inflammation is based on findings indicating that the removal of either caspase-8 or Fadd in IECs is sufficient to induce necroptosis, leading to spontaneous ileitis and/or colitis.^205^^,^^217^ Later, the expression of a secreted form of interferon-λ in mice resulted in a loss of PCs from intestinal tissues, via STAT1 and MLKL, controlled by caspase-8.^218^ In 2019, Lehle et al^219^ described patients with inherited caspase-8 deficiency that developed intestinal inflammation and dysregulated immunity. In further studies, an excessive activation of RIPK3 in the inflamed tissues of individuals with IBD and a positive correlation between necroptosis and the severity of the disease among individuals with IBD were noted.^205^^,^^220^^,^^221^ In a previous investigation conducted by Lee et al,^222^ it was observed that the administration of a RIPK3 inhibitor significantly reduced the expression of proinflammatory cytokines in peripheral blood mononuclear cells of individuals with UC and in mice exhibiting colitis induced by DSS. In addition to the previously mentioned modes of programmed cell death, autophagy-associated cell death has also been linked to IBD.

## Conclusions

An emerging hypothesis suggests that IBD may arise from dysregulated communication between IECs, the immune system, and the microbiota. Differentiated IECs possess several defense mechanisms, including cell-cell contacts or the secretion of antimicrobial peptides, and mucus production to maintain barrier function and to counteract pathogens and harmful agents. The segmental expression and heterogeneity of TJ protein serve unique roles in maintaining intestinal health, but their functions are compromised in IBD. Changes in expression and localization result in site-specific alterations in paracellular barrier and channel functions, ultimately leading to enhanced paracellular permeability. Regulated cell death, the extrusion, and replacement of IECs is of utmost importance to ensure barrier maintenance. Numerous genetic factors have been identified that regulate cell death and are associated with the development of IBD. Mesenchymal cells and PCs in the surrounding of stem cells provide the optimal environmental conditions for the development of new IECs, with dramatic consequences if, for example, Wnt or other growth factors are dysregulated. The recognition of microbes and the adequate response to commensals and pathogens ensure that the epithelial cell layer is not destroyed. In IBD, many of these functions are dysregulated and lead to an imbalance between cell death and cell renewal and thus to gaps in the epithelium, which in turn is associated with the penetration of pathogens. The treatment of IBD is currently only possible to a limited extent and further analysis of influencing factors and signal transmission to the epithelium is necessary for the development of additional therapeutic options.
